# Trends in Respiratory Symptoms of Firefighters Exposed to the World Trade Center Disaster: 2001–2005

**DOI:** 10.1289/ehp.0800291

**Published:** 2009-02-11

**Authors:** Mayris P. Webber, Jackson Gustave, Roy Lee, Justin K. Niles, Kerry Kelly, Hillel W. Cohen, David J. Prezant

**Affiliations:** 1 Montefiore Medical Center, Albert Einstein College of Medicine, Bronx, New York, USA; 2 Fire Department of the City of New York, Bureau of Health Services, Brooklyn, New York, USA; 3 Albert Einstein College of Medicine, Bronx, New York, USA

**Keywords:** disaster medicine, firefighters, gastroesophageal reflux disease (GERD), occupational medicine, rescue workers, respiratory symptoms, World Trade Center

## Abstract

**Background:**

Respiratory symptoms, either newly reported after the World Trade Center (WTC) disaster on 11 September 2001 (9/11) or increased in severity, have been well documented in WTC-exposed workers and New York City residents. However, considerable uncertainty exists over the persistence of symptoms.

**Objectives:**

In this study, our goals were to describe trends in post-9/11 respiratory and gastro-esophageal reflux disease (GERD) symptoms in WTC-exposed firefighters and to examine symptom progression in the cohort that completed both year 1 and year 4 questionnaires.

**Methods:**

We analyzed questionnaire responses from 10,378 firefighters in yearly intervals, from 2 October 2001 to 11 September 2005, defining exposure based on arrival time at the WTC site. For the cohort of 3,722 firefighters who completed the two questionnaires, we also calculated exposure duration summing months of work at the site.

**Results:**

In cross-sectional analyses, the prevalence of dyspnea, wheeze, rhinosinusitis, and GERD remained relatively stable, whereas cough and sore throat declined, especially between 1 and 2 years post-9/11. We found a dose–response relationship between arrival time and symptoms in all years (*p* < 0.01). Logistic models of symptoms at year 4 in the cohort demonstrated independent effects of earlier arrival and longer work duration: each additional month of work increased the odds of symptoms 8–11%.

**Conclusions:**

Protracted work exposures increased the odds of respiratory and GERD symptoms 4 years later. In most large disasters, exposures may be unavoidable during the rescue phase, but our data strongly suggest the need to minimize additional exposures during recovery and cleanup phases.

The collapse of the World Trade Center (WTC) on 11 September 2001 (9/11) and subsequent recovery efforts released large amounts of particulate dust, combustion particles, gases, fumes, and other noxious materials, some of which will remain incompletely characterized [Banauch et al. 2006;Centers for Disease Control and Prevention (CDC) 2002a; Edelman et al. 2003; Landrigan et al. 2004; Lioy et al. 2002; McGee et al. 2003; Mendelson et al. 2007], particularly the gaseous component (Lioy et al. 2002). After the initial dust cloud settled, fires continued to be a major source of ongoing airborne contamination for months after the attack. The Fire Department, City of New York (FDNY), operated a continuous rescue/recovery effort at the site that lasted until July 2002 (Fireman et al. 2004; Prezant et al. 2002) and involved > 15,000 rescue workers [firefighters and emergency medical service (EMS) workers].

In the first 6 months after 9/11, the Bureau of Health Services (BHS) of FDNY identified 332 firefighters with “World Trade Center cough,” defined as a persistent cough that developed after exposure to the site and that was severe enough to require extensive medical leave (Prezant et al. 2002). In the same report, a range of frequent coexistent symptoms were identified, including dyspnea, wheeze, nasal congestion/drip, and acid reflux, that were consistent with asthma, rhinosinusitis, and gastro-esophageal reflux disease (GERD). Increased bronchodilator responsiveness and bronchial hyperreactivity were often noted in this and other studies (Banauch et al. 2003, 2005a; Prezant et al. 2002). A later study involving 12,079 FDNY rescue workers documented a decline in pulmonary function during the first year post-9/11 that was 12 times that found pre-9/11 (Banauch et al. 2006). An exposure–response gradient, based on arrival time at the WTC (Prezant et al. 2002), was consistently demonstrated for symptoms, bronchodilator responsiveness, bronchial hyperreactivity, and declines in pulmonary function. Respiratory symptoms, either newly reported post-9/11 or increased in severity, have also been documented in other exposed workers (Herbert et al. 2006; Herbstman et al. 2005; Salzman et al. 2004; Tao et al. 2007; Wheeler et al. 2007) and residents (CDC 2002b; Lin et al. 2005; Reibman et al. 2005; Szema et al. 2004).

Considerable uncertainty exists over the persistence of respiratory symptoms among WTC-exposed individuals because of a lack of long-term follow-up data. One earlier study reported on respiratory symptoms at three time points (pre-9/11, while working at the site, and 10–31 months post-9/11), but the first two time points were collected retrospectively and therefore were potentially subject to recall bias (Herbert et al. 2006). Another study evaluated symptom persistence at 1 and 20 months post-9/11 but focused exclusively on lower respiratory symptoms (LRS) in a convenience sample of 471 police officers (Buyantseva et al. 2007). The present study is the first to report the evolution of WTC respiratory symptoms in a large, highly exposed, homo geneous (similar fire-fighting job tasks) group (*n* = 10,378), with prospective follow-up over the first 4 years. We describe temporal trends in the prevalence of post-9/11 aerodigestive symptoms [upper respiratory symptoms (URS), LRS, and GERD] through 11 September 2005. We further analyzed the progression of symptoms in those firefighters (*n* = 3,722) that completed questionnaires during both the first and fourth years, thereby enabling assessment of four symptom patterns: early onset/resolved, early onset/persistent, delayed onset, and asymptomatic initially and after 4 years (Buyantseva et al. 2007).

## Methods

Since 1997, the FDNY BHS has performed periodic health evaluations on FDNY members approximately every 18 months; these evaluations include physician examinations and, since 2001, self-administered health questionnaires. The questionnaires are programmed on touch-screen computers, with trained personnel available to answer questions. Participation in the study required written informed consent and was approved by the Institutional Review Board of Montefiore Medical Center.

### Study participants

The original sample consisted of 14,380 firefighters and EMS workers who were hired before 25 July 2002 (the date the WTC site closed). We excluded 1,636 firefighters who arrived after day 14 or were never present (because of demographic differences between them and earlier arrivals); 369 who did not complete questionnaires; and 1,997 EMS workers (because of differences in their job tasks and because they had less stringent preemployment health requirements). The final sample for cross-sectional analysis consisted of 10,378 firefighters.

### Data sources

We obtained demographics from the FDNY employee database. Symptoms, exposure status, mask/respirator use, and smoking history were collected from the questionnaires.

### Symptom prevalence

The questionnaire asked participants about LRS and URS: “Since the disaster, have you had any of the following new or worsening respiratory symptoms?” For LRS, possible answer choices included “no respiratory symptoms,” “wheezing,” “shortness of breath,” and “daily cough”; for URS, possible answers included “no nose or throat symptoms,” “nasal drip,” “nasal congestion,” “sore throat,” and “hoarse throat with change or loss of voice.” Multiple answers were allowed. We estimated GERD symptoms based on a positive response to “chest tightness or pain” or “stomach upset or heartburn.” Follow-up questionnaires asked participants if they had any of the above symptoms, and affirmative answers were qualified as to their presence during “the last 4 weeks.” We obtained information on symptoms pre-9/11 from the participant’s first post-9/11 questionnaire, which asked: “Prior to the disaster did you commonly suffer from any of the following: daily cough, nasal congestion or drip, wheeze, shortness of breath, chest tightness or pain.” Multiple answers were allowed.

### WTC exposure

The FDNY-WTC exposure intensity index (Prezant et al. 2002) categorized exposure based on first arrival at the WTC site as follows: group 1 (the most severely exposed) arrived on the morning of 9/11 and were present during the tower collapses; group 2 arrived during the afternoon of 9/11; group 3 arrived on day 2 (12 September 2001); and group 4 (least exposed) arrived on days 3–14.

In addition to arrival group, we created two duration variables. The first used information from questions in which participants reported which months they worked at the site, on or off duty, from September 2001 through July 2002. We used the sum of the number of months participants worked on the site as a continuous variable in multivariate models. We created the second variable, based on both work duration and mask/respirator use, by multiplying each month the participant reported working at the site by 1.0, 0.75, or 0.25, depending on the reported mask/respirator use frequency of “never,” “rarely,” or “mostly” during that month (Wheeler et al. 2007). The sum of the number of mask-use–weighted months was tested as a continuous variable in multivariate models.

### Smoking history

“Current smokers” reported smoking cigarettes during any year post-9/11. “Former smokers” reported smoking before 9/11 but did not report current smoking in any post-9/11 questionnaire. “Never smokers” consistently reported not smoking pre- and post-9/11.

### Time periods

In this study we include data from 17,447 questionnaires analyzed cross-sectionally in 1-year periods based on the date administered: year 1, 2 October 2001 to 11 September 2002; year 2, 12 September 2002 to 11 September 2003; year 3, 12 September 2003 to 11 September 2004; and year 4, 12 September 2004 to 11 September 2005. Within each year, if persons completed more than one questionnaire, we used data only from the earliest one.

### Cohort for analysis of symptom patterns over time

To identify symptom patterns and their relative frequency, we also analyzed a cohort (*n* = 3,722) that completed questionnaires during both the first and fourth years according to four reported symptom patterns: early onset/resolved, early onset/persistent, delayed onset, and asymptomatic initially and at 4years (Buyantseva et al. 2007).

### Statistical analyses

Bivariate analyses of categorical variables used the chi-square test with odds ratios (ORs) and 95% confidence intervals (95% CIs). We assessed continuous variables using the *t*-test or analysis of variance. In cross-sectional analyses, we used marginal logistic regression models fitted with generalized estimating equations to assess differences in the annual prevalence of symptoms by arrival group, and McNemar’s test for trend to assess the relation of arrival group to symptoms.

In the cohort, we tested the linear trend of symptom prevalence over time by arrival group using the Cochran-Armitage test for trend. We used multiple logistic regression analyses with backward elimination to predict outcomes of any LRS, URS, or GERD at follow-up. Variables tested in all models included age and years of FDNY service on 9/11, arrival group, duration of work at the WTC site in months and weighted for mask/respirator use, symptoms before 9/11, symptoms reported on initial questionnaire, smoking status (current, former, or never), and elapsed time between initial and follow-up questionnaires. We individually tested three interaction terms in each model: arrival group and smoking history, arrival group and months of work, and mask/respirator use and months of work. Variables remained in the model based on a *p*-value of ≤ 0.05 and assessment of their impact on other variables in the model. Goodness of fit was assessed using the Hosmer-Lemeshow test. Data were analyzed using SAS, version 9.1 (SAS Institute Inc., Cary, NC).

## Results

We collected 17,447 questionnaires from 10,378 WTC-exposed firefighters over the 4-year period from 2 October 2001 to 11 September 2005. During the study, firefighter compliance with scheduled periodic evaluations every 18 months, including questionnaire completion, was 85%. Most participants were male (99.8%), were white (93.6%), and never smoked (73.1%). The number of participants in each year of the serial cross-sectional analyses was 8,920 in year 1; 1,197 in year 2; 2,889 in year 3; and 4,441 in year 4. By arrival group, 16.2% (1,683) arrived during the morning on 9/11, 63.7% (6,611) during the afternoon of 9/11, 11.7% (1,215) on day 2, and 8.4% (869) on days 3–14. The overall mean (± SD) duration of work at the WTC site was 4.4 ± 2.8 months, which differed significantly by arrival group: 4.7 ± 3.0, 4.4 ± 2.8, 3.9 ± 2.5, and 3.3 ± 2.3 months for arrival groups 1–4, respectively (*p* < 0.0001). Comparing the group of 10,378 with the cohort of 3,722, we found small statistically significant differences in mean age (39.2 years vs. 37.3 years; *p* < 0.01) and percent Caucasian (93.6% vs. 94.7%; *p* < 0.01), whereas percent male (99.8%) and percent arrival group 1 (16.2%) were the same.

### Prevalence of symptoms in cross-sectional analyses

Before 9/11, participants had rarely reported LRS: frequent cough was reported by 4.1%, dyspnea by 2.5%, and wheeze by 1.2%. In the first year (2 October 2001 to 11 September 2002), the most common LRS was frequent cough, reported by 54.2%. By year 2, the rate of frequent cough declined to 16.9%, remaining close to that level to affect 15.7% during year 4. In contrast, dyspnea and wheeze showed little change: dyspnea was reported by 40% during year 1 and 38.8% during year 4, and wheeze was reported by 34% throughout all 4post-9/11 years.

**Figure 1:**
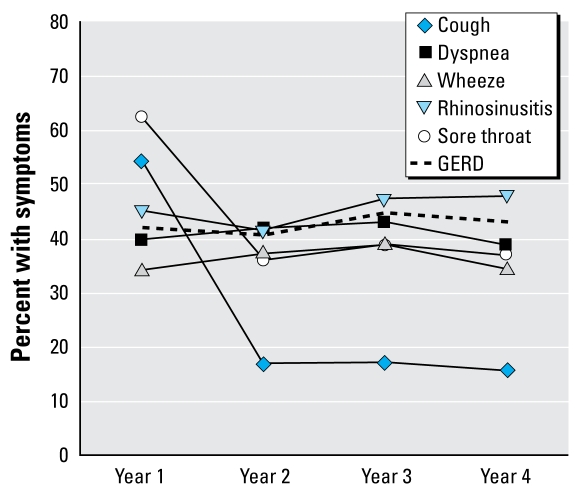
Trends in the prevalence of symptoms in 10,378 firefighters from 2001 through 2005.

Before 9/11, reports of URS were also rare, with frequent sore throat reported by 3.2% and frequent rhinosinusitis by 4.4%. During year 1, the most common respiratory symptom was sore throat, reported by 62.4%. By year 2, the rate of sore throat declined to 36.0%, plateauing to affect 37.0% in year 4. In contrast, rhinosinusitis showed little change, varying from 45.1% to 47.8% during years 1 and 4, respectively. Before 9/11, symptoms consistent with GERD were reported by 5.2%. GERD symptoms were reported by 41.8% during year 1 and remained between 40% and 45% during all 4post-9/11 years (Figure 1).

### Prevalence by arrival group in cross-sectional analyses

**Figure 2:**
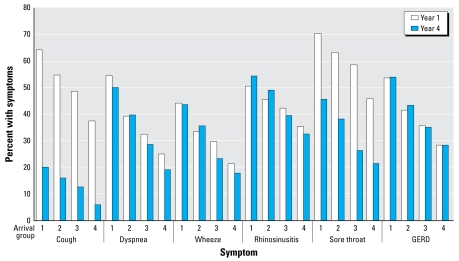
Trends in the prevalence of symptoms in 10,378 firefighters during year 1 (2001–2002) and year 4 (2004–2005) by arrival group.

For all symptoms, earlier arrival was associated with higher prevalence in all years (all *p* < 0.01). For cough, dyspnea, wheeze, sore throat, and rhinosinusitis, those in arrival groups 3 and 4 experienced a greater proportion of decline over time compared with changes in earlier arrival groups (Table 1, Figure 2).

### Symptom progression in the cohort

A total of 3,722 firefighters completed both year 1 and year 4 questionnaires. In year 1, the mean (± SD) number of reported symptoms per person was 2.6 ± 2.0, which significantly declined to 2.2 ± 2.0 (*p* < 0.0001) in year 4.

On the initial questionnaire, 64.1% reported one or more LRS, 69.7% one or two URS, and 38.4% GERD. At year 4, the prevalence of any LRS declined significantly to 49.5%, largely attributable to the 69.0% decline in cough, because both dyspnea and wheeze significantly increased from 35.2% to 39.4% and from 28.9% to 34.6%, respectively (*p* < 0.001 for both). Similarly, we found a significant decline (*p* < 0.001) in any URS to 57.3%, primarily attributable to a 37.4% decline in sore throat, because rhinosinusitis significantly increased from 44.1% to 48.7% (*p* < 0.001). The prevalence of GERD also increased from 38.4% to 43.8% (*p*< 0.001).

### Factors associated with symptom patterns in the cohort

We explored the relationship between arrival group and symptom patterns in the cohort with year 1 and year 4 questionnaires (*n* = 3,722). Earlier arrival group was consistently related to symptom persistence for LRS, URS, and GERD. Comparing earliest arriving participants (arrival group 1) with all others, arrival group 1 members were more likely to have LRS (OR = 1.8; 95% CI, 1.5–2.2), URS (OR = 1.5; 95% CI, 1.3–1.8), and/or GERD (OR = 1.9; 95% CI, 1.5–2.2) at year 4. This held true for each individual symptom as well. For LRS, persistent cough (OR = 1.7; 95% CI, 1.3–2.1), persistent dyspnea (OR = 2.0; 95% CI, 1.7–2.4), and persistent wheezing (OR = 1.8; 95% CI, 1.5–2.3) were all more likely among arrival group 1 members. A similar pattern was apparent for those with persistent URS: persistent rhinosinusitis (OR = 1.3; 95% CI, 1.1–1.6) and persistent sore throat (OR = 1.6; 95% CI, 1.3–1.9). In contrast, asymptomatic status was consistently related to later arrival status (*p*< 0.0001; Table 2).

The prevalence of smoking in the cohort was 13.5%, 11.1%, and 75.4% for current, former, and never smokers, respectively. Current and former smokers were generally overrepresented among those with persistent symptoms. We also carried out analyses comparing persons with persistent symptoms with those who recovered. We found that current smoking compared with never smoking was associated with persistent wheeze (OR = 1.5; 95 CI, 1.1–2.1), cough (OR = 1.5; 95% CI, 1.1–2.0), and GERD (OR = 1.6; 95% CI, 1.2–2.3). Former smoking compared with never smoking was not significantly associated with individual symptoms but was associated with persistent LRS (OR = 1.3; 95% CI, 1.0–1.8) and URS (OR = 1.3; 95% CI, 1.0–1.7).

### Multivariate analyses

Multivariate logistic regression models in the cohort predicting symptoms at year 4, either persistent or delayed onset were carried out separately for LRS, URS, and GERD outcomes. Arrival group, initial symptoms, age on 9/11, and months of work (either modified by mask/respirator use or unmodified) were independently associated with symptoms at follow-up in all models. We used the unmodified duration variable because results did not differ from those using the modified variable. Elapsed time between year 1 and year 4 questionnaires remained significant only in the LRS model. Three interaction terms—months of work and arrival group, smoking and arrival group, and months of work and mask/respirator use—were not statistically significant (all *p* > 0.05). The overlap between LRS, URS, and GERD was apparent, especially in the model predicting GERD, where the addition of terms for initial LRS and rhinosinusitis symptoms greatly improved the model fit. All models satisfied Hosmer-Lemeshow goodness of fit tests (Tables 3–5).

## Discussion

In this study we describe the prevalence of respiratory symptoms in a well-characterized group of 10,378 WTC-exposed firefighters who worked, on average, four times longer at the WTC site (Wheeler et al. 2007) and were followed more than twice as long (4 years) as workers in most previous reports (Buyantseva et al. 2007; Tao et al. 2007; Wheeler et al. 2007). We found that cough, previously characterized in air pollution studies as the most sensitive indicator of lower respiratory insult (Dockery and Pope 1994), similarly served as a sentinel indicator in WTC-exposed firefighters. In our study, cough was one of the earliest-appearing and earliest-resolving symptoms, declining sharply between 1 and 2 years post-9/11 to affect 16% of fire fighters by the study’s end. Despite this sharp decline, cough rates during the final period were approximately four times their pre-9/11 rate. Other LRS rates after 9/11 remained relatively constant, and by study’s end, wheeze and dyspnea were reported at 28.6 and 15.5 times their pre-9/11 rates, respectively.

For URS, the prevalence of sore throat declined by 41%, whereas rhinosinusitis symptoms increased by 6%. The final rates were 10.7 and 10.6 times their pre-9/11 rates, respectively. GERD symptoms increased by 3.2% during the study, with a final rate 8.2 times its pre-9/11 prevalence. We are confident that comparing rates during the final study period with pre-9/11 rates is valid even though the latter were collected retrospectively at the first post-9/11 questionnaire, because these data are comparable with information collected during FDNY periodic medical evaluations obtained pre-9/11.

Analyses of the cohort of 3,722 enabled us to examine reported-symptom progression in the group of firefighters who completed questionnaires in both the first and fourth postexposure years, thereby allowing differentiation between symptom patterns of persistence, delayed onset, resolution, and never symptomatic at year-4 follow-up. It also allowed us to explore the evolution of symptoms within individuals, as opposed to tracking change over time in the larger population. Multivariate analyses of the cohort data yielded one of our major findings. After adjusting for other variables in the model, we found that each month worked at the WTC site increased the odds of symptoms at follow-up by 11% for both LRS and GERD and by 8% for URS. At the maximum duration of 10 months, the odds of symptoms in year 4 were 2.8 times greater for LRS and GERD and 2.2 times greater for URS. In fact, 10 months of work at the site was much more strongly associated with year 4 symptoms than even the earliest arrival time. Most studies have not reported on work duration (Banauch et al. 2005a; Buyantseva et al. 2007; Fireman et al. 2004; Landrigan et al. 2004; Oppenheimer et al. 2007). Of those that did, one found an association with new-onset asthma (Wheeler et al. 2007); another reported associations with respiratory symptoms at 20 months (Buyantseva et al. 2007), although it did not control for early arrival at the WTC site; and a third found no association with post-9/11 symptoms (de la Hoz et al. 2008). Our data strongly suggest that the impact of protracted WTC work exposure was of critical importance and should be considered in future disaster plans.

Another important finding of the cohort analyses was that cough resolution did not mean that persons were free of LRS. Of those with resolved cough, half (50.9%) reported persistent or delayed-onset dyspnea, wheeze, or both at follow-up. Furthermore, because cough may arise from either lower or upper respiratory problems, we looked at URS in those with resolved cough and found that 61.3% of former coughers reported sore throat, rhinosinusitis symptoms, or both at follow-up. us, our data confirm our clinical impression that persons with resolved cough should not be considered symptom-free (Buyantseva et al. 2007). Similarly, among persons with URS, those with resolved sore throat had a 36.3% prevalence of rhino sinusitis symptoms, persistent or delayed in onset, during year 4.

Consistent with initial reports by this group (Banauch et al. 2005a, 2005b; Prezant et al. 2002) and others (de la Hoz et al. 2008; Herbert et al. 2006), we found considerable overlap of LRS, URS, and GERD symptoms. Adding terms for symptoms of the other respiratory groups at the initial period improved all multivariate models. Describing cough as an LRS simplifies analyses but may misclassify cough, which can also be due to rhinosinusitis symptoms (congestion/drip), sore throat, and/or GERD (Irwin et al. 1993). In fact, we found that about 30% of the cohort reported at least one LRS, one URS, and GERD symptoms during both initial and follow-up times (data not shown).

Cigarette smoking is a modifiable risk factor increasing the likelihood of LRS and GERD by approximately 50% at follow-up. This is but one of many reasons for supporting aggressive efforts to promote nonpunitive tobacco cessation programs and counseling services, available without cost at FDNY since 9/11 (Bars et al. 2006).

This study’s primary limitation was its lack of access to information about treatment, which precluded estimating the effects of treatment on reported symptoms over time. We also acknowledge that both arrival group and months of work are only crude measures of exposure to the WTC site, which would better be measured by knowing specific hours and days an individual worked on-site. However, our arrival group measure was independently corroborated by others who described the intensity of outdoor exposures based on days post-9/11 (Lioy et al. 2002). Symptom reporting could have been biased because of issues related to workers’ compensation, retirement disability, and civil litigation; it could also have been affected by recall bias or by the wording of our questions, which asked about symptoms since 9/11. However, in a previous study (Banauch et al. 2006) we reported that objective spirometric measurements correlated with clinical complaints, and each additional respiratory symptom was associated with an additional decrement in forced expiratory volume in 1 sec. Despite potential limitations, we found significant exposure–response gradients for symptoms at all time points. We believe the strengths of this study outweigh its limitations. First, we examined secular trends in > 10,000 firefighters and were able to study symptom progression in the cohort of 3,722 based on their questionnaire responses at both years 1 and 4. Compared with other published studies, this represents a larger group of highly exposed individuals, all with similar firefighting job tasks, and the most years of follow-up. Second, we asked about the prevalence of symptoms pre-9/11, which enabled us to compare pre- and post-9/11 symptoms in individuals whom we knew to be extremely healthy pre-9/11. Finally, we explored the data in several ways. For example, we initially selected the cohort based on persons with two or more visits at least 1 year apart to increase the number of participants, but ultimately selected only those with first- and fourth-year questionnaires to allow the maximum time for symptoms to resolve, either spontaneously or through treatment. Using either cohort did not change findings.

## Conclusion

We found that cough and sore throat were the most sensitive indicators of initial respiratory insult early after 9/11, but that the other symptoms (wheeze, dyspnea, rhinosinusitis, and GERD) were more sensitive indicators at follow-up. In year 4, even cough, the symptom with the greatest decline rate, was still reported at four times the pre-9/11 level, and the other symptoms were reported at levels from 8.2 to 28.6 times their pre-9/11 rates. We found a significant exposure–response gradient based on arrival time for all symptoms. In the cohort we found that each month worked at the site conferred a substantial and highly significant increase in the odds of symptoms at follow-up. For LRS, 5 months of work exposure conferred a risk equivalent to arriving at the WTC site during the morning of 9/11, and 10 months of work was associated with an almost 3-fold risk of symptoms at follow-up. The importance of this finding cannot be overemphasized. In any disaster, exposures will be difficult to avoid in the first weeks when the potential for successful rescues are time-limited and the environment may be difficult to control. However, during subsequent recovery and cleanup phases, it is reasonable to expect appropriate protection from potential environmental hazards; our data strongly suggest the need to develop and implement strategies that include guidelines for respirator use to minimize additional exposures during these phases.

## Correction

In the original manuscript published online, affiliations were incorrect for M.P.W. and J.K.N. They have been corrected here.

## Figures and Tables

**Table 1:** Annual prevalence of symptoms in 10,378 firefighters by arrival group (%)^a^

		Year^b^					
Symptom	Arrival group	1 (*n* = 8,920)	2 (*n* = 1,197)	3 (*n* = 2,889)	4 (*n* = 4,441)	Percent change, years 1–4^c^	*p*-Value^d^
Cough	1	64.3	25.9	24.3	20.0	−68.9	< 0.0001

	2	54.8	16.1	16.9	16.1	−70.6	< 0.0001
	3	48.4	13.7	12.6	12.7	−73.8	< 0.0001
	4	37.4	8.4	10.7	6.0	−84.0	< 0.0001

Dyspnea	1	54.4	57.7	54.3	50.1	−7.9	0.554

	2	39.2	42.3	43.2	39.6	1.0	0.002
	3	32.5	31.3	34.9	28.7	−11.9	0.273
	4	25.0	20.0	26.6	19.2	−23.2	0.269

Wheeze	1	44.2	51.2	48.4	43.5	−1.5	0.090

	2	33.7	36.5	38.7	35.5	5.6	< 0.0001
	3	29.7	31.3	32.7	23.2	−21.9	0.034
	4	21.5	22.1	23.7	17.9	−16.7	0.514

Rhinosinusitis	1	50.6	50.2	53.9	54.4	7.4	0.193

	2	45.5	41.8	48.4	49.1	7.9	< 0.0001
	3	42.4	37.4	40.9	39.6	−6.6	0.416
	4	35.5	24.2	31.6	32.5	−8.5	0.079

Sore throat	1	70.4	48.8	48.9	45.6	−35.1	< 0.0001

	2	63.2	35.5	39.2	38.2	−39.6	< 0.0001
	3	58.5	32.1	28.6	26.3	−55.1	< 0.0001
	4	45.9	18.9	26.6	21.5	−53.1	< 0.0001

GERD	1	53.7	51.2	53.6	53.9	0.4	0.645

	2	41.5	39.4	45.9	43.2	4.1	< 0.0001
	3	35.6	39.7	35.5	35.0	−1.7	0.847
	4	28.3	30.5	27.7	28.5	0.7	0.781

a *p*-Values all < 0.01, by McNemar’s test for trend between symptom prevalence and arrival group.

b Individuals in each year may not be the same.

c Calculated as (year 4 – year 1)/(year 1) × 100.

d *p*-Values from marginal logistic regression model fitted with generalized estimating equations, with an exchangeable within-individual correlation.

**Table 2:** Symptom progression [no. (%)] by arrival group in the cohort (*n* = 3,722) at year 4.

	Arrival group					
Symptom pattern	1 (*n* = 603)	2 (*n* = 2,504)	3 (*n* = 384)	4 (*n* = 231)	Total (*n* = 3,722)	*p*-Value^a^
Cough						

Early onset/recover	260 (43.1)	1,014 (40.5)	136 (35.4)	67 (29.0)	1,477 (39.7)	< 0.0001
Persistent	104 (17.3)	297 (11.9)	39 (10.2)	9 (3.9)	449 (12.1)	< 0.0001
Delay onset	20 (3.3)	105 (4.2)	13 (3.4)	7 (3.0)	145 (3.9)	0.77
Asymptomatic	219 (36.3)	1,088 (43.5)	196 (51.0)	148 (64.1)	1,651 (44.4)	< 0.0001

Wheeze						

Early onset/recover	64 (10.6)	284 (11.3)	48 (12.5)	25 (10.8)	421 (11.3)	0.64
Persistent	156 (25.9)	446 (17.8)	42 (10.9)	11 (4.8)	655 (17.6)	< 0.0001
Delay onset	103 (17.1)	445 (17.8)	51 (13.3)	35 (15.2)	634 (17.0)	0.15
Asymptomatic	280 (46.4)	1,329 (53.1)	243 (63.3)	160 (69.3)	2,012 (54.1)	< 0.0001

Dyspnea						

Early onset/recover	81 (13.4)	319 (12.7)	43 (11.2)	22 (9.5)	465 (12.5)	0.09
Persistent	207 (34.3)	558 (22.3)	63 (16.4)	19 (8.2)	847 (22.8)	< 0.0001
Delay onset	95 (15.8)	441 (17.6)	54 (14.1)	31 (13.4)	621 (16.7)	0.23
Asymptomatic	220 (36.5)	1,186 (47.4)	224 (58.3)	159 (68.8)	1,789 (48.1)	< 0.0001

Any LRS						

Early onset/recover	138 (22.9)	602 (24.0)	107 (27.9)	50 (21.7)	897 (24.1)	0.58
Persistent	315 (52.2)	1,016 (40.6)	112 (29.2)	45 (19.5)	1,488 (40.0)	< 0.0001
Delay onset	40 (6.6)	245 (9.8)	42 (10.9)	29 (12.6)	356 (9.6)	0.004
Asymptomatic	110 (18.2)	641 (25.6)	123 (32.0)	107 (46.3)	981 (26.4)	< 0.0001

Rhinosinusitis						

Early onset/recover	91 (15.1)	395 (15.8)	63 (16.4)	36 (15.6)	585 (15.7)	0.71
Persistent	202 (33.5)	722 (28.8)	95 (24.7)	39 (16.9)	1,058 (28.4)	< 0.0001
Delay onset	126 (20.9)	520 (20.8)	63 (16.4)	44 (19.1)	753 (20.2)	0.17
Asymptomatic	184 (30.5)	867 (34.6)	163 (42.5)	112 (48.5)	1,326 (35.6)	< 0.0001

Sore throat						

Early onset/recover	184 (30.5)	771 (30.8)	128 (33.3)	60 (26.0)	1,143 (30.7)	0.60
Persistent	229 (38.0)	748 (29.9)	80 (20.8)	36 (15.6)	1,093 (29.4)	< 0.0001
Delay onset	49 (8.1)	208 (8.3)	26 (6.8)	22 (9.5)	305 (8.2)	0.93
Asymptomatic	141 (23.4)	777 (31.0)	150 (39.1)	113 (48.9)	1,181 (31.7)	< 0.0001

Any URS						

Early onset/recover	132 (21.9)	583 (23.3)	104 (27.1)	51 (22.1)	870 (23.4)	0.35
Persistent	332 (55.1)	1,182 (47.2)	148 (38.5)	61 (26.4)	1,723 (46.3)	< 0.0001
Delay onset	57 (9.5)	272 (10.9)	44 (11.5)	38 (16.5)	411 (11.0)	0.008
Asymptomatic	82 (13.6)	467 (18.7)	88 (22.9)	81 (35.1)	718 (19.3)	< 0.0001

GERD						

Early onset/recover	80 (13.3)	335 (13.4)	42 (10.9)	28 (12.1)	485 (13.0)	0.35
Persistent	217 (36.0)	628 (25.1)	71 (18.5)	27 (11.7)	943 (25.3)	< 0.0001
Delay onset	110 (18.2)	461 (18.4)	70 (18.2)	47 (20.4)	688 (18.5)	0.59
Asymptomatic	196 (32.5)	1,080 (43.1)	201 (52.3)	129 (55.8)	1,606 (43.2)	< 0.0001

a *p*-Values from Cochran–Armitage test for trend.

**Table 3:** Multiple logistic regression models in the cohort of 3,722 firefighters [OR (95% CI)] for LRS at year 4.

Variable	OR (95% CI)
Any URS, year 1 questionnaire	1.45 (1.21–1.73)
GERD, year 1 questionnaire	1.27 (1.07–1.51)
Cough, year 1 questionnaire	1.62 (1.38–1.90)
Dyspnea, year 1 questionnaire	2.34 (1.94–2.82)
Wheeze, year 1 questionnaire	1.91 (1.59–2.30)
Current smoking	1.56 (1.26–1.93)
Age on 9/11 (years)	1.03 (1.02–1.04)
Arrival group 1^a^	1.66 (1.16–2.37)
Arrival group 2^a^	1.49 (1.08–2.04)
Arrival group 3^a^	1.13 (0.77–1.64)
Months of work at WTC site	1.11 (1.08–1.14)
Days between questionnaires	0.999 (0.998–1.00)

a Reference is arrival group 4 (arrival at WTC site 3–14 days post-9/11).

**Table 4:** Multiple logistic regression models in the cohort of 3,722 firefighters [OR (95% CI)] for URS at year 4.

Variable	OR (95% CI)
Any LRS, year 1 questionnaire	1.53 (1.29–1.80)
Rhinosinusitis, year 1 questionnaire	2.06 (1.77–2.39)
Sore throat, year 1 questionnaire	1.73 (1.48–2.04)
Arrival group 1^a^	1.63 (1.17–2.28)
Arrival group 2^a^	1.40 (1.04–1.88)
Arrival group 3^a^	1.09 (0.77–1.55)
Age on 9/11 (years)	1.011 (1.00–1.02)
Months of work at WTC site	1.08 (1.05–1.11)

a Reference is arrival group 4 (arrival at WTC site 3–14 days post-9/11).

**Table 5:** Multiple logistic regression models in the cohort of 3,722 firefighters [OR (95% CI)] for GERD at year 4.

Variable	OR (95% CI)
Any LRS, year 1 questionnaire	1.71 (1.45–2.03)
Rhinosinusitis, year 1 questionnaire	1.20 (1.03–1.39)
GERD, year 1 questionnaire	3.48 (2.98–4.05)
Current smoking	1.49 (1.22–1.83)
Arrival group 1^a^	1.49 (1.05–2.11)
Arrival group 2^a^	1.15 (0.84–1.58)
Arrival group 3^a^	1.03 (0.71–1.49)
Age on 9/11 (years)	1.02 (1.01–1.03)
Months of work at WTC site	1.11 (1.08–1.14)

a Reference is arrival group 4 (arrival at WTC site 3–14 days post-9/11).
